# Adoptive cell therapy with autologous tumor infiltrating lymphocytes and low-dose Interleukin-2 in metastatic melanoma patients

**DOI:** 10.1186/1479-5876-10-169

**Published:** 2012-08-21

**Authors:** Eva Ellebaek, Trine Zeeberg Iversen, Niels Junker, Marco Donia, Lotte Engell-Noerregaard, Özcan Met, Lisbet Rosenkrantz Hölmich, Rikke Sick Andersen, Sine Reker Hadrup, Mads Hald Andersen, Per thor Straten, Inge Marie Svane

**Affiliations:** 1 CCIT, Center for Cancer Immune Therapy, Department of Hematology, Copenhagen University Hospital, Herlev, Denmark; 2 Department of Oncology, Copenhagen University Hospital, Herlev, Denmark; 3 Department of Biomedical Sciences, University of Catania, Catania, Italy; 4 Department of Plastic Surgery, Copenhagen University Hospital, Herlev, Denmark

**Keywords:** Adoptive cell therapy, Tumor infiltrating lymphocytes, Malignant melanoma, Low-dose Interleukin-2

## Abstract

**Background:**

Adoptive cell therapy may be based on isolation of tumor-specific T cells, e.g. autologous tumor infiltrating lymphocytes (TIL), in vitro activation and expansion and the reinfusion of these cells into patients upon chemotherapy induced lymphodepletion. Together with high-dose interleukin (IL)-2 this treatment has been given to patients with advanced malignant melanoma and impressive response rates but also significant IL-2 associated toxicity have been observed. Here we present data from a feasibility study at a Danish Translational Research Center using TIL adoptive transfer in combination with low-dose subcutaneous IL-2 injections.

**Methods:**

This is a pilot trial (ClinicalTrials.gov identifier: NCT00937625) including patients with metastatic melanoma, PS ≤1, age <70, measurable and progressive disease and no involvement of the central nervous system. Six patients were treated with lymphodepleting chemotherapy, TIL infusion, and 14 days of subcutaneous low-dose IL-2 injections, 2 MIU/day.

**Results:**

Low-dose IL-2 considerably decreased the treatment related toxicity with no grade 3–4 IL-2 related adverse events. Objective clinical responses were seen in 2 of 6 treated patients with ongoing complete responses (30+ and 10+ months), 2 patients had stable disease (4 and 5 months) and 2 patients progressed shortly after treatment. Tumor-reactivity of the infused cells and peripheral lymphocytes before and after therapy were analyzed. Absolute number of tumor specific T cells in the infusion product tended to correlate with clinical response and also, an induction of peripheral tumor reactive T cells was observed for 1 patient in complete remission.

**Conclusion:**

Complete and durable responses were induced after treatment with adoptive cell therapy in combination with low-dose IL-2 which significantly decreased toxicity of this therapy.

## Background

The incidence of malignant melanoma is increasing and every year more than 68.000 Americans will be diagnosed with this disease [1]. Most patients are cured after resection of the primary tumor but for approximately 15% the disease will metastasize. Once disseminated, malignant melanoma has a very poor prognosis with a 5-year survival rate of less than 15% [1]. Chemotherapy and radiotherapy have limited efficacy, and treatments with curable potential have been restricted to Interleukin(IL)-2 and Interferon(IFN)-α which has high toxicity, and low response rates. Recently, several new treatment modalities have been approved for the treatment of metastatic melanoma. This includes the anti-CTLA-4 antibody, Ipilimumab [2,3] and the B-RAF inhibitor, Vemurafenib [4], along with new drugs in the pipeline, e.g. PD-1 antibodies [5,6] and MEK-inhibitors [7]. Still, a large majority of the patients will eventually progress on these therapies leaving a need for additional treatment modalities with curable potential.

Adoptive cell therapy (ACT) with tumor infiltrating lymphocytes (TILs) was pioneered by Rosenberg and co-workers and has shown very promising results with high response rates and long-term survivors [8-10]. Despite this, the treatment has only been applied in a few centers around the world. The complicated procedure of establishing and expanding TIL cultures is one reason but also the toxicity of high-dose IL-2 limits the implementation of this therapy. IL-2 is administered to support further proliferation and persistency of the transferred T cells but whether high doses of IL-2 is required for induction of clinical response is not known. In a previous study [11], varying doses of IL-2 were administered together with antigen-specific T cells, but the optimal dose of IL-2 administered with TILs remains to be determined.

This pilot study was designed to investigate the feasibility of ACT using low-dose subcutaneous IL-2 injections instead of high dose intravenous IL-2. The objectives were to examine the toxicity profile, to evaluate the possibility of inducing complete and long-lasting clinical responses and to investigate potential immune parameters. Results from this trial, including safety data, clinical and immunological outcome are reported in the following.

## Materials and methods

### Patients

Patients between ages of 18–70 years with metastatic malignant melanoma were included. Further inclusion parameters were disease in progression (according to response evaluation criteria in solid tumors (RECIST)), measurable disease according to RECIST, at least one resectable metastasis at a minimum of 1 cm^3^, and performance status (PS) ≤ 1. Furthermore, patients had to be seropositive for EBV antigen and negative for hepatitis B and C infection and HIV. Exclusion criteria were significant heart or lung disease, autoimmune disease, other neoplastic tumors within the last 5 years, treatment with steroids, or involvement of the central nervous system.

All patients signed a written informed consent before entering the study.

### Treatment and trial design

The trial was approved by the medical agencies, the ethical committees and the data agency, and was conducted in accordance with the Helsinki declaration and good clinical practice as described by Danish law (ClinicalTrials.gov ID: NCT00937625).

Patients were admitted to hospital at day −8 and a central venous catheter was applied. A lymphodepleting chemotherapy regimen consisting of Cyclophosphamide, 60 mg/kg/d day −7 to −6 and fludarabine phosphate, 25 mg/m2/d at day −5 to −1 administered as previously described [10]. Prophylactic antiemetics with palonosetron, aprepitant, and Domperidone were given together with pantoprazole. At day 0 autologous TILs were infused intravenously followed by 14 days of subcutaneous IL-2 injections, 2 MIU, starting the same evening.

Patients were treated prophylactically with trimethoprim, sulfamethoxazole, and acyclovir from the beginning of treatment and 6 months thereafter and with fluconazole during the leucopenic period.

Clinical response was monitored with a computed tomography (CT) or positron emission tomography (PET)/CT scan 8 weeks after T cell infusion and assessed according to RECIST 1.0. All scans were reviewed by an independent radiologist at the Hospital who was blinded to previous descriptions.

### TIL culturing and expansion

Tumor material was obtained by excision of subcutaneous nodules (5 patients) or lymph nodes (6 patients). The tumor specimen was excised aseptically and transported in AIM-V (Invitrogen, Nærum, Denmark) media containing Fungizone. The TIL culturing method has been adapted from Dudley et al. [12] and has previously been described [13]. Briefly, the tumor sample were cut into 1–2 mm fragments and placed in 24 well-culture plates (Nunc, Roskilde, Denmark) together with 2 ml of culture medium (90% RPMI 1640 (Invitrogen), 10% heat inactivated Human AB serum (Sigma-Albricht, St. Louis, MO, USA), IL-2 6000 IU/ml (Aldesleukin, Novartis, Basel, Suisse), penicillin, streptomycin and fungi zone (Bristol-Myers Squibb, Lyngby, Denmark). Cells were split into 2–3 wells when cell concentration in 1 well exceeded 1.5 x 10^6^ cells/ml. Each fragment and the following TIL cultures were kept separate during further expansion. Cell counting and viability testing were stained with tryphan blue and analyzed by microscopy.

TIL cultures with 90% viability and more than 60 x 10^6^ cells were frozen or transferred for further expansion in the rapid expansion protocol (REP). Sterility testing and microbiological control were performed on all TIL cultures before freezing or expansion.

TIL bulk cultures were selected for rapid expansion based on proliferative capacity (the fastest growing cultures) and the highest percentage of CD3+, CD45RO+, CCR7+/− and CD8+ phenotypic markers. TILs were co-cultured with allogeneic irradiated peripheral blood monocytes (PBMCs) serving as feeder cells together with culture medium AIM-V, anti-CD3 antibody (OKT-3, Cilag AG, Suisse), and IL-2 in upright T175 flasks. At day 14 the expansion rate had increased by more than 1000 fold and the cells were harvested and infused in the lymphodepleted patient.

### Immunological assays

Fluorochrome-conjugated antibodies for flow cytometry were the following: CD3, CD4, CD8, CD27, CD45RO, CCR7, IFN-γ, TNF-α, and CD107a (BD, Brøndby, Denmark).

The infusion product and PBMCs obtained before and after treatment from each patient were tested for reactivity against short-term cultured autologous melanoma cell lines (when available), generated from the same specimen used for TIL generation by serial passage of adherent cells [14], or against a panel of 4 to 8 HLA-A matched allogeneic melanoma cell lines. In addition, infusion products were also tested for reactivity against a panel of tumor-derived peptides. The following techniques have been applied.

### Intracellular cytokine staining (ICS)

A flow cytometry-based assay was performed in order to depict the production of cytokines when cells from the infusion product were co-cultured with 1) tumor cell lines, as previously described [14] or with 2) mRNA-transfected autologous DCs: *1) Co-culture with tumor cell lines*: TILs were cultured for 5 hours at 37°C with 5% CO_2_ in air in the presence or absence of melanoma cells at an effector/target ratio of 3:1. *2) Co-culture with mRNA-transfected autologous DCs*: One patient (patient 11) had previously been included in a clinical phase I protocol at our center (ClinicalTrials.gov ID: NCT00978913) in which DCs transfected with mRNA encoding p53, survivin or hTERT were evaluated in patients with metastatic breast cancer or malignant melanoma (Additional file 1). TILs from this patient were cultured for 5 hours with i) the DC-vaccine, ii) autologous DCs transfected with p53, survivin and hTERT mRNA, iii) autologous DCs transfected with single mRNA and iv) autologous DCs transfected with mock mRNA (i.e. negative control) at an effector/target ratio of 10:1. Staphylococcal Enterotoxin B (SEB) (Sigma-Aldrich, 5 μg/ml final concentration) was used as positive control. In selected analyses anti-CD107a (BD, 0.03 ug/ml final concentration) was added at the beginning of the incubation. GolgiPlug (Sigma-Aldrich) was added at a dilution of 1:1000 after the first hour of incubation. After 4 additional hours cells were washed twice with PBS, stained with Fixable Viability Dye (Ebiosciences) and with antibodies directed to surface markers. Cells were washed one additional time, fixed overnight, permeabilized and subsequently stained with antibodies for intracellular cytokines. Cells were analyzed using a BD FACSCanto II flow cytometer. At least 1 x 10^5^ TILs or lymphocytes were acquired. Analysis was performed with BD FacsDiva Software. Responding cells were defined as those who stained double positive for IFN-γ and TNF-α.

### ELIspot assays

An IFN-γ ELIspot assay was performed to quantify the number of TILs responding to 1) tumor-associated peptides, as previously described [14,15] or to 2) mRNA transfected autologous DCs.

Briefly, nitrocellulose bottomed 96 well plates (Multiscreen MAIP N45, Millipore, Copenhagen, Denmark) were coated with IFN-γ capture antibody (1-DIK, Mabtech, Nacka Strand, Sweden). The wells were washed, blocked with X-VIVO 15 medium and TILs were added in triplicates at different cell concentrations. *1) TILs responding to tumor-associated peptides:* TILs were cultured for 4 hours at 37°C with 5% CO_2_ in air with or without tumor-associated peptides (final concentration 5μM). *2) TILs responding to mRNA-transfected autologous DCs:* TILs were cultured for 4 hours at 37°C with 5% CO_2_ in air with i) the DC-vaccine, ii) autologous DCs transfected with p53, survivin and hTERT mRNA, iii) autologous DCs transfected with single mRNA and iv) autologous DCs transfected with mock mRNA (i.e. negative control). After addition of secondary biotinylated antibody (7-B6-1-Biotin, Mabtech) and Streptavidin-enzyme conjugates (Streptavidin-ALP, Mabtech), the enzyme substrate nitro blue tetrazolium/5-bromo-4-chloro-3-indolyl phosphate (NBT/BCIP, Mabtech) was added to each well and the reactions were stopped with tap water. Spots were counted with the ImmunoSpot Series 2.0 Analyzer (CTL Analyzers). A positive response was defined as more than twice the background and at least 50 spots/well.

### MHC-multimer staining

To screen for reactivity against a large panel of melanoma associated peptides we used flow cytometry based detection of MHC multimer binding T cells by a combinatorial encoding technique [16].

Peptides were purchased from Pepscan (Pepscan Presto BV, Lelystad, Netherlands) and dissolved to 10 mM in DMSO. Recombinant MHC heavy chains and β2microglobulin light chain were produced in Escherichia coli and refolded with conditional ligands, as described by Hadrup et al. [17]. Specific peptide-MHC complexes were produced by UV-mediated peptide exchange of conditional ligands [17,18]. The conditional ligands were synthesized as previously described [18-20]. MHC multimers were generated using 8 different streptavidin (SA)-fluorochrome conjugates (SA-PE, SA-APC, SA-PE-Cy7 (BioLegend, San Diego, CA, USA), SA-quantum dot (Qdot)585, SA-Qdot605, SA-Qdot625, SA-Qdot655 and SA-Qdot705 (Invitrogen)). Each peptide-MHC multimer was generated in two different colors, which allows a two-color coding upon staining of specific T cells, as described [16]. This leads to 28 unique two-color codes, of which 27 are functional. These are used for staining of 27 T cell populations in 1 sample. Eight panels were prepared for analysis of 175 melanoma-associated T cell epitopes (10 HLA-A1, 146 HLA-A2, 11 HLA-A3, 3 HLA-A11 and 5 HLA-B7 epitopes) [21]. Eighteen virus-derived T cell epitopes were included as positive control of the method.

All T cell stainings were performed on cryopreserved material. Up to 10^6^ cells per sample were stained with 1 MHC multimer panel for 15 min at 37°C and 5% CO_2_. Next, cells were stained with anti-CD8-Alexa Fluor 700 (Biolegend), dump channel antibodies (CD4-, CD14-, CD16-, CD19-FITC (BD) and CD40-FITC (AbD SeroTec, Oxford, UK)) and a dead cell marker (LIVE/DEAD Fixable Near-IR, Invitrogen) for 30 min on ice. Final staining volume was 100 μl. Subsequently, cells were washed twice with PBS containing 2% fetal bovine serum (FBS) and resuspended in 50 μl PBS containing 2% FBS. Data acquisition was performed on an LSR-II flow cytometer (BD), and data analysis was carried out using FACSDiva software (BD). Responses were defined as a minimum of 10 spots and a minimum of 0.002% of CD8^+^ T cells. All responses were verified in a validation screen, using different color-codes for the given MHC multimer specificity.

### Statistical analysis

Survival was defined as the time from initiation of treatment until death or last date of follow-up (March 1^st^, 2012). Time from treatment initiation until exclusion from the trial due to disease progression was defined as time to progression (TTP).

## Results

### Patient characteristics

Eleven patients with metastatic malignant melanoma were enrolled from June 2009 till June 2011. Six of the 11 included patients were treated according to the protocol. All patients were previously treated with high-dose IL-2 according to an intravenous decrescendo regimen previously described by Keilholz et al. [22]. Demographic data for these 6 patients are depicted in Table 1.

**Table 1:** Patient demographics

**Patient number**	**Age(years)**	**Sex**	**HLA- type**	**PS**	**Previous treatments**	**Tumor burden (cm)**	**AJCC stage**	**Metastatic sites**
1	60	F	A2	0	IL2/INF, brain surgery	3.9	M1c	LN, brain^a^
2	47	M	A2/A3	0	IL2/IFN, DC-vac, abdominal surgery	22.8	M1c	LN, intestines
3	62	M	A3/A11	0	IL2/IFN, DC-vac	15.5	M1c	SC, lung, stomach, gall bladder
6	36	M	A2	0	IL2/IFN	8.6	M1c	LN, liver, bone
7	61	M	A2/A24	0	IL2/INF, CD137, DC-vac, brain surgery	19.8	M1c	LN, lung, bone, brain^a^
11	41	M	A1/A3	0	IL2/INF, Ipilimumab, DC-vac	3.5	M1a	LN, SC

PS: performance status, AJCC: American Joint Committee on Cancer, M:male, F:female, IL2/INF:Interleukin-2/Interferon-α (according to the treatment regimen described by Keilholz et al. ref. 20), DC-vac: dendritic cell vaccine (experimental treatment), CD137:CD137 antibody (experimental treatment), LN: lymphnode metastasis, SC: subcutaneuous metastasis. Tumor burden was defined as the sum of all measurable lesions (>1 cm in longest diameter) up to a maximum of 10 lesions (according to RECIST 1.0). ^a^Brain metastases were surgically removed before inclusion in the protocol.

From 1 of the 11 patients TIL cultures were not established. Two patients developed large symptomatic brain metastases during the time of TIL culturing and 2 patients had rapid disease progression after tumor resection. They were therefore excluded from the trial before treatment initiation. Thus, 5 patients were included in the trial and had tumor resected but were not treated.

### Clinical results

Of the 6 treated patients, 2 patients achieved a complete response (CR), 2 patients had stable disease (SD) and 2 patients progressed rapidly (Table 2).

**Table 2:** Treatment characteristics and clinical outcome

	**Phenotype and functionality of the infusion products**							**Clinical outcome**		
**Patient number**	**Infused cells**	**CD8**^**+**^	**CD45RO**^**+**^	**CCR7**^**+**^	**CD27**^**+**^	**Reactivity**^a^	**Total number of responding CD8**^**+**^**T cells infused**	**Response**	**TTP**	**OS**
	**(x 10**^**10**^**)**	**(%)**	**(%)**	**(%)**	**(% of CD8)**	**(%)**	**(x 10**^**6**^**)**	**(RECIST)**	**(mo)**	**(mo)**
**1**	**2.9**	**94**	**99.7**	**7.5**	**6.1**	**28.8**^**#**^	**6460**	**CR**	**30+**	**30+**
2	1.8	92	97.4	4.9	5.2	1.0^#^	175	PD	2	7
3	2.0	95	99.4	6.1	0.5	0.0^#^	7	SD	4	11.5
6	0.3	47	98.4	3.9	1.2	0.9*	41	PD	2	4.6
7	1.3	88	89	31.8	3.4	0.4^#^	44	SD	5	11
**11**	**7.5**	**95**	**99**	**0.6**	**8.1**	6.0*	**4377**	**CR**	**10+**	**10+**

Table showing phenotypic and functional characteristics of the infusion products as well as clinical outcome for the 6 treated patients.

^a^Reactivity meaning tumor-specific CD8+ T cells in % of infused TILs. Reactivity was calculated by intracellular cytokine staining measuring the percentage of CD8+ T cells expressing TNF-α and IFN-γ after co-culture with *autologous tumor cell lines or ^#^allogeneic melanoma cell lines. For patients tested against several allogeneic melanoma cell lines reactivity against the cell line that resulted in the highest reactivity has been shown (for further details please see Additional file 2). RECIST: Response Evaluation Criteria in Solid Tumors, TTP: time to progression, OS: overall survival, mo: months, CR: complete response, SD: stable disease, PD: progressive disease.

Patient 1 had a brain metastasis surgically excised prior to inclusion in this trial. At time of inclusion the patient had disseminated disease limited to lymph nodes in the pelvis and 1 of these were removed for the preparation of TILs. Because of post-surgery complications with abscess formation and a peripheral deep venous thrombosis cells were frozen until the patient had recovered. After therapy this patient had slow regression of the metastases leading to a negative PET/CT scan 1½ years after treatment. This patient is continuously in complete remission 30 months after treatment.

**Figure 1:**
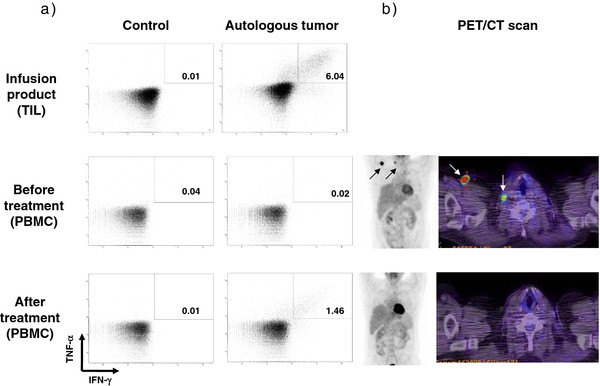
**Immune and clinical evaluation of patient 11.a**) FACS plot from intracellular cytokine staining showing the percentage of interferon (IFN)-γ and tumor necrosis factor (TNF)-α producing CD3^+^CD8^+^T cells after incubation with autologous tumor cell lines or Staphylococcal Enterotoxin B (control). First row showing reactivity of tumor infiltrating lymphocytes (TIL) from the infusion product, second and third row showing reactivity of peripheral blood monocytes (PBMC) 1 week before infusion of TILs and 3 weeks after. **b**) PET/CT scan from 1 week before infusion of TILs and 8 weeks after infusion of TILs. Arrows outlining the measurable disease.

Patient 11 was initially included in a dendritic cell vaccination protocol but because of large metastases on the neck and cheek the patient underwent palliative plastic surgery. Tumor material was sent to the laboratory and TIL cultures were established and frozen for later use. Four months later the patient progressed on the initial treatment and was subsequently included in this protocol. Eight weeks after T-cell infusion there were no sign of disease and previous PET-positive lesions were negative (Figure 1b). This patient is in ongoing complete remission 10 months after therapy.

Patient 3 had SD on the 1^st^ evaluation scan but 4 months after T-cell therapy an increase in tumor burden of 26% led to exclusion from the protocol. Also patient 7 had SD but developed a new metastasis 5 months after therapy and was therefore excluded due to progressive disease (PD). Patient 2 and 5 had PD at 1^st^ evaluation 8 weeks after therapy.

In total, the 6 patients have a median TTP of 8.2 months (range 2–30 months) and an overall survival (OS) of 12 months (range 5–30 months) with 2 ongoing complete responses (Table 2).

### Toxicity

In general, toxicity grade 3–4 (according to Common Terminology Criteria for Adverse Events (CTCAE) v. 3.0) was related to the lymphodepleting chemotherapy with gastrointestinal symptoms, fatigue and low blood cell counts. All patients had a grade 2 anemia and received blood transfusions, 1 patient had thrombocytopenia and received platelet transfusions and all patients had grade 4 leucopenia, neutropenia and lymphopenia (Table 3).

**Table 3:** Toxicity

	**Grade 1**	**Grade 2**	**Grade 3**	**Grade 4**
Performance status	1	4	1	
				
Fatigue	2	3	1	
Leucopenia				6
Neutropenia				6
Lymphopenia				6
Thrombopenia			2	1
Anemia		6		
Nausea	1	4		
Diarrhea	2	2	1	
Vomiting	2		1	
Infections	2		1	
Alopecia		6		
Dermatitis	2	1		
Allergic reaction	3			
Low sodium levels	2		3	1

No grade 3–4 events were associated with Interleukin-2 treatment. Grade is referring to Common Terminology Criteria for Adverse Events (CTCAE) v. 3.0. Digits in the table are referring to number of patients with the given adverse event.

Notably, very low sodium levels were observed in the first patients during administration of Cyclophosphamide. We found that restriction of oral water intake during infusion of Cyclophosphamide could control the sodium level in the following patients.

The infusion of TILs led to fever and chills for most of the patients. Two patients reacted to the infusion of TILs with high blood pressure and tachycardia. Symptoms were treated with morphine, antihistamines and oxygen and relieved after a few minutes.

Low-dose IL-2 was well tolerated and all planned injections without dose reductions were given. As expected, patients developed fever during treatment with IL-2 injections and were consequently treated with antibiotics. Other side effects were chills a few hours after injection, nausea and fatigue, although none of these exceeded grade 2 toxicity. Nausea and fatigue were assessed to be related to the previously administered chemotherapy.

### TIL characteristics

Approximately 18 cultures were initiated from each patient and 3–5 of these gave rise to a culture with sufficient growth for further expansion. REP was started at an average cell count of 18 x 10^6^ (range 11–30 x 10^6^) cells after a mean time of 32.5 days (range 24–42 days) in culture. In average 26 x 10^9^ (range 3.4 – 74.7 x 10^9^) TILs were infused in each patient (Table 2).

Phenotype of the infused cells showed that the majority of cells were effector memory T cells expressing (CD3 (97-100%) and CD45RO (89-100%) and low expression of CCR7 (0.6-32%)). The percentage of CD8 T cells ranged from 47-95%, and between 0.5-8.1% of the CD8 cells were CD27 positive (Table 2).

Reactivity of the infusion products was tested using several different techniques; including ICS, IFN-γ ELIspot assay and MHC multimer staining.

Infused T cells were tested for production of different cytokines (TNF-α and IFN-γ) when co-cultured with autologous melanoma cell lines (2 patients) or a panel of HLA-A matched allogeneic cell lines (4 patients) (Additional file 2). Reactivity of the TILs against autologous tumor from patient 11 is depicted in Figure 1a, upper row. The 2 clinical responding patients (patient 11 tested against an autologous cell line and patient 1 tested against an allogeneic cell line) had higher reactivity than the non-responding patients (Table 2). Interestingly, a more than 80 fold higher absolute number of tumor-specific CD8^+^ cells in the infusion product was observed among the 2 responding patients compared to the 4 non-responders (5418 x 10^6^ vs. 67 x 10^6^ CD8^+^ T cells (mean values)) (Table 2). Reactivity against the tumor cell lines were confirmed for the 2 responding patients by direct IFN-γ ELIspot analyses (data not shown).

Reactivity against a selected panel of peptide epitopes from well-characterized melanoma antigens was tested with direct IFN-γ ELIspot analyses (Table 4a). In these analyses we found reactivity against 23-60% of the tested peptides. In addition, we tested for the presence of CD8 T cells recognizing a large panel of 173 peptides, representing all published epitopes of relevance for melanoma (the full peptide list can be found in Andersen RS et al. [21]). To screen for reactivity against this large panel of melanoma associated peptides a combinatorial encoding technique was applied [16] (Examples of MHC multimer stainings have been presented in Additional file 3). Using this technique, we confirmed only 3 of the responses detected by ELIspot and detected another 8 responses towards a limited set of peptides from this large peptide library (Table 4b). Most of the T cell responses detected were of low frequency, in concordance with previous published data [21,23], and even this large peptide library is limited in describing the epitope-specificity of the autologous and allogeneic tumor cell recognition observed. There were no correlation between peptide specific reactivity and clinical response.

**Table 4:** T-cell responses detected in TIL infusion products

**a) IFN-γ ELIspot analyses**							
**Patient ID**		**1**	**2**	**3**	**6**	**7**	**11**
HLA-type		A2	A2/A3	A3, A11	A2	A2	A1, A3
A2	Bcl-2_WLD_	NR	43	-	NR	301	-
	Bcl-x_RIA_	NR	165	-	NR	197	-
	hTERT_ILA_	180	456	-	363	462	-
	CB9L2_ALY_	232	135	-	293	441	-
	CB9 204_ILI_	488	502	-	325	408	-
	NY-ESO 1_SLL_	180	564	-	158	279	-
	MAGE A1_KVL_	NR	57	-	NR	190	-
	MAGE A3_FLW_	91	67	-	118	250	-
	SUR1M2_LML_	ND	96	-	ND	349	-
	SUR9_ELT_	ND	62	-	ND	274	-
	MART-1_ELA_	ND	324*	-	193*	NR	-
	gp100_ITD_	ND	106	-	ND	ND	-
A3	Bcl-x_RIA_	-	284	111	-	-	59
	SUR18K10_RIS_	-	ND	112	-	-	NR
	SUR53_DLA_	-	96	ND	-	-	82
	Rho C (nat)_RAG_	-	ND	114	-	-	NR
	Rho C (mod)_RLG_	-	ND	103	-	-	NR
	TAG_RLS_	-	ND	ND	-	-	42*
Number of responses/total screened		5/15	14/24	4/9	6/15	9/15	3/13
Background		99	46	86	130^a^/92^b^	131	29
**b) MHC multimer staining**							
**Patient ID**		**1**	**2**^**c**^	**3**	**6**	**7**	**11**^**d**^
HLA-type		A2	A2, A3, B7	A3, A11	A2	A2	A1, A3
A2	MART-1_ELA_	0.013	0.07*	-	1.10*	0.008	-
	gp100_YLE_	0.010	NR	-	10.0	NR	-
	gp100_KTW_	0.010	NR	-		NR	-
	gp100_ITD_	0.002	NR	-	0.018	NR	-
	GnT-V_VLP-9_	0.015	NR	-	NR	NR	-
A3	TAG_RLS_	-	NR	NR	-	-	0.60*
Number of responses/total screened		5/146	1/162	0/14	3/146	1/146	1/21

**a)** IFN-γ ELIspot analyses; Responses are defined as number of spots per 1 x 10^5^ T cells, except for patient 7 (2.5 x 10^4^ cells) and patient 11 (1.25 x 10^4^ cells). Background level shown at the bottom of the table has been subtracted in the shown responses. ^a/b^: two different assays have been performed wherefore two background values are present.

**b)** MHC multimer staining; Antigen-specific T cells are given in percentage of CD8^+^ T cells. Examples of MHC multimer stainings can be seen in Additional file 3. ^c^The TILs analyzed were frozen two days after infusion. ^d^The TILs analyzed were frozen one day after infusion.

Last row indicates the number of detected responses out of the total number of peptides screened. NR: no reactivity, ND: reactivity testing not done, -: not tested due to HLA-discrepancy.

*Indicating responses found in both methods. nat: natural, mod: modified.

### Immune monitoring

PBMCs from before and after treatment were tested by ICS for reactivity against autologous tumor cell lines when available (patient 11), otherwise the allogeneic melanoma cell lines against which the infusion product had shown highest reactivity were used (patient 1, 2, 3 and 7). No increase in baseline reactivity was seen, except for patient 11 who had no activity when tested at baseline but developed a response 1 week after T cell infusion which was confirmed after 3 weeks (Figure 1a). This patient had a complete clinical response to the treatment (Figure 1b).

### Re-activation of previously activated T cells from a patient treated with a dendritic cell (DC) vaccination

**Figure 2:**
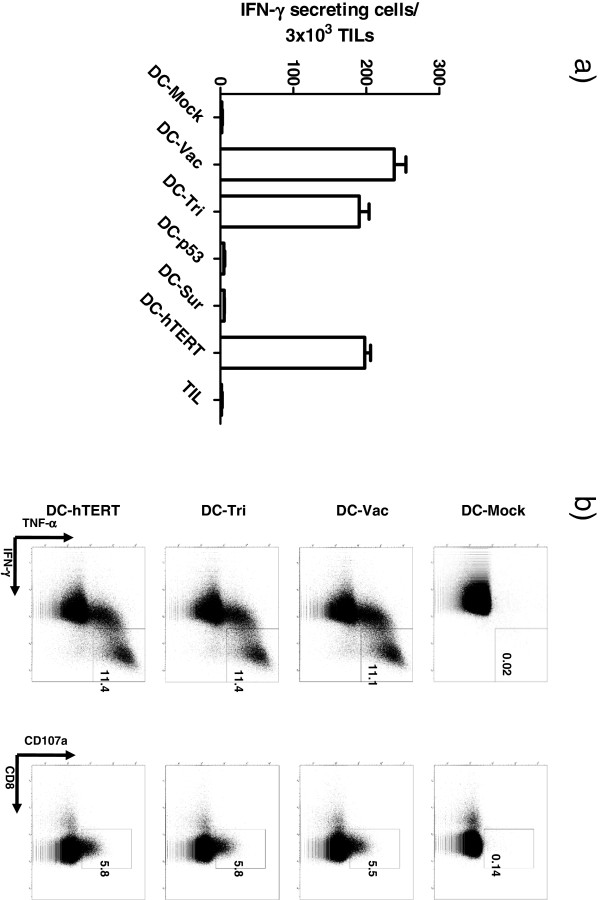
**Infusion product tested for reactivity against a dendritic cell vaccine (patient 11).a**) IFN-γ ELIspot analyses; Responses are defined as number of IFN-γ secreting cells per 3 x 10^3^ TILs. **b**) Intracellular cytokine staining; percentage of T cells staining double positive for IFN-γ and TNF-α (first column) or for CD8 and CD107a (second column) DC-mock: mock-transfected dendritic cell (negative control), vac: vaccine, tri: triple transfected, sur: survivin, hTERT: human Telomerase Reverse Transciptase, TIL: tumor infiltrating lymphocytes.

One patient (patient 11) had previously been included in a clinical phase I trial where DCs transfected with mRNA encoding p53, survivin or hTERT were evaluated in patients with metastatic breast cancer or malignant melanoma (Engell-Noerregaard et al. trial ongoing, see Additional file 1 for further information). Accordingly, we questioned whether the infusion product could elicit immune responses to the mRNA transfected DC-vaccine. For this purpose, TILs from patient 11 were stimulated with either the DC-vaccine or DCs transfected with triple mRNA and vaccine-specific TILs were subsequently analyzed with ELIspot IFN-γ release assay and ICS. Using this approach, we were able to detect IFN-γ TIL reactivity against the DC-vaccine and transfected DCs in both the ELIspot and ICS assay (Figure 2). Further analysis using single mRNA-transfected DCs as target revealed that the TIL response was predominantly against hTERT-transfected DCs (Figure 2a). This was consistent with the responding TILs detected using the ICS assay (Figure 2b). No response was detected when TILs were stimulated with p53, survivin or mock-transfected DCs (Figure 2).

## Discussion

Herein we report the results from 6 patients treated with lymphodepleting chemotherapy, autologous TILs and very low-doses of IL-2. This trial shows that it is possible to induce complete and long-lasting responses even with the use of low-dose IL-2 that significantly reduced the toxicity of therapy. Today, ACT is only implemented in few clinical centers, but if high-dose IL-2 was not required, it could be possible to offer this therapy more widely and to more patients. With this said, this is a pilot trial including only 6 patients, and whether the doses of IL-2 affects the clinical response rate warrants further study. Thus, larger clinical studies are needed to confirm whether high response rates can be maintained with the use of lower doses of IL-2 in combination with ACT.

Investigations on the use of low-dose IL-2 in an ACT setting have been performed by others. Yee et al. [24] demonstrated that adoptively transferred T cell clones targeting melanoma-associated antigens could persist in vivo in response to very low doses of IL-2. Two other groups [25,26] have shown that Melan-A-specific CD8^+^ T cells were able to induce long-lasting responses in metastatic melanoma patients and that transferred cells persisted and even expanded in vivo. In these studies the infusion of T cells was followed by subcutaneous injections of low-dose IL-2 and/or IFN-α suggesting that low-doses of cytokines might well be sufficient to prolong survival of the transferred cells and induce objective clinical responses. This is further underlined by the results from Verdegaal et al. [27] who reported on a clinical study transferring blood derived tumor-specific T- cells into metastatic melanoma patients in combination with low-dose IFN-α still observing long lasting clinical responses. A recently published retrospective report from Ullenhag and colleagues [28] described a cohort of patients treated with ACT and a low dose IL-2 regimen with 2.4 MIU/m^2^ once a day continuing until progression. Long lasting response in 1 of the patients was reported; however, continuous treatment with IL-2, even in low-doses, might significantly interfere with quality of life for those patients achieving durable responses. Besides, it is questionable whether long-term IL-2 treatment is necessary for continuous tumor control [8,9].

Only 6 of 11 included patients who underwent tumor resection with the intent to treat actually received treatment. In this study, patients were treated with TILs from selected individual cultures resulting in a culturing time (including REP) of about 7–8 weeks. Treating patients with unselected young TILs can decrease the production time to 4–5 weeks [8,29]. Also, the use of engineered cells for costimulatory enhancement during TIL expansion has been described to accelerate TIL growth [30] and hereby decrease the time from surgery to treatment. Accelerated preparation time might reduce the drop-out rate, as 4 out of 5 patients who were not treated in this trial were excluded due to deterioration of performance and/or the appearance of brain metastases awaiting the treatment. Furthermore, methods to improve the reliability of TIL production have been examined in order to diminish the number of patients not treated due to insufficient TIL growth [30,31]. Validation and implementation of these processes will reveal if this can further increase the fraction of treated patients.

The infusion product for each patient was tested for reactivity against a large panel of melanoma antigens. Screening for reactivity against different peptides showed some responses but these were unable to fully explain the anti-tumor reactivity observed. The limited peptide reactivity is in line with recent data, showing recognition of only a small fraction of the described melanoma-associated peptides and predominantly very low frequency of antigen specific T cell populations [23,32]. Furthermore, only few of the responses detected by IFN-γ ELIspot could be confirmed in the assessment by MHC multimers. However, it may be speculated that part of this discrepancy relies to the fact that the majority of the “self-reactive” T cells posses a very low affinity to the MHC possibly insufficient for MHC-multimer binding, but sufficient for stimulation of IFN-γ secretion upon addition of excess amounts of peptide. In addition, a number of reports have shown that IFN-γ secretion after stimulation with short peptides may result from CD4 T cells (Eckhart Kämpgen, Erlangen, Germany, personal communication), and the performed IFN-γ ELIspot analyses did not discriminate between CD4 and CD8. Nevertheless, the TIL infusion product consisted of mostly CD8 T cells (Table 2) wherefore this would be able to explain only a limited part of the differences in reactivity.

Conclusions on correlation between clinical response and patient demographics or treatment characteristics are not possible with 6 treated patients. However, the 2 responding patients’ characteristics differ from the non-responding patients. Both responding patients had gone through extensive surgery and had, at the time of treatment initiation, only limited disease burden, suggesting that debulking of tumor-mass may play a role for the outcome of TIL therapy using low-dose IL-2. Also, a high percentage of CD8 T cells and a high number of infused TILs were characteristic for the responding patients (Table 2). Even though several patients, including non-responding patients had a high percentage of CD8 T cells in the infusion product this might, together with other factors such as high reactivity against melanoma cell lines, increase the possibility of obtaining a clinical response. Interestingly, we found that a high absolute number of tumor-reactive T cells in the infusion product were more than 80 times higher for patients with a clinical response than for non-responders.

Correlations between clinical response and patient demographics or treatment characteristics have been intensively investigated by others. In ACT trials using high dose IL-2 no correlation between tumor burden and clinical response has been observed previously [9] and whether our findings are due to the lower amount of IL-2 given or whether it might just be a coincidence considering the low number of patients treated can not be concluded in this pilot study. Besser et al. [8] also found a correlation between the percentage of CD8^+^ T cells in the infusion product and clinical response as well as between the number of infused cells and clinical response. Longer telomer length, a high percentage of CD8^+^CD27^+^ TILs and persistency of the infused cells in the circulation have also been shown to correlate with clinical response [33] wherefore new methods resulting in shorter culturing time (young TILs) has emerged [8].

Future initiatives on how to increase the tumor-reactivity of infused TILs should be considered. We have recently shown that IFN-γ is able to increase the immunogenicity of melanoma cells thereby restoring the responsiveness in otherwise unresponsive T cells in clinical TIL products (Donia M et al. accepted for publication, J Invest Dermatol, 2012) and IFN-α has in a previous study been shown to be able to induce clinical responses in combination with ACT [27]. Also, other agents, such as BRAF inhibitors have been shown to have immune modulating potential [34-36]. Thus, the use of these agents in combination with ACT could have the potential to enhance immunogenicity of tumor cells and thereby increase the fraction of tumor-specific T cells in the TIL product capable of killing tumor cells.

A high percentage of vaccine-specific T cells were found in the infusion product from a patient previously treated with an mRNA transfected DC vaccine. We hypothesize that vaccine-specific T cells were induced during the DC vaccination but were not able to overcome immunosuppressive mechanisms and therefore did not give rise to a clinical significant anti-tumor response. When T cells were activated and expanded ex-vivo and re-infused into the lymphodepleted patient a clinical response could be established. Further analyses will be performed to clarify whether the vaccine-specific cells are indeed induced during vaccination and whether these cells equal the tumor reacting cells.

## Conclusions

In conclusion, we demonstrate that durable complete responses can be achieved using ACT and short duration of low-dose IL-2. The much less toxic regimen simplifies the clinical setting of this therapy making it more attractive for other centers to establish. Also, the possibility of an association between the absolute number of tumor-reactive T cells infused and clinical response is shown. This knowledge could be used in the future search for optimizing ACT, with new trials focusing on increasing the tumor-sensitivity to T cell mediated killing as well as the number of potent tumor-reactive T cells.

Our findings of cancer vaccine-specific T cells in the infusion product from a patient who subsequently achieved complete response support the idea of inducing anti-tumor T cells by a cancer vaccine. The technique for subsequent expansion in vitro have been established in a preclinical setting [37] and will be incorporated in a clinical trial initiated in the near future. Also, we have initiated a new trial for metastatic melanoma patients using young-TIL ACT in combination with intermediate doses of IL-2 (ClinicalTrials.gov ID: NCT00937625) with the purpose of defining the most optimal dose of IL-2 for the use in a larger randomized clinical trial.

## Abbreviations

ACT: Adoptive cell transfer; TIL: Tumor infiltrating lymphocytes; IL-2: Interleukin-2; IFN: Interferon; TNF: Tumor necrosis factor; RECIST: Response evaluation criteria in solid tumors; CTCAE: Common terminology criteria for adverse events; CT: Computed tomography; PET: Positron emission tomography; REP: Rapid expansion protocol; PBMC: Peripheral blood monocytes; HLA: Human leucocyte antigen; DC: Dendritic cell; SEB: Staphylococcal enterotoxin B; ELIspot: Enzyme-linked immunosorbent spot; CR: Complete response; PR: Partial response; SD: Stable disease; PD: Progressive disease; TTP: Time to progression; OS: Overall survival.

## Competing interests

The authors declare that they have no competing interests.

## Authors’ contributions

EE participated in conception and design, acquisition of data, and analysis and interpretation of data as well as drafting the manuscript. TZI, LEN and LRH participated in acquisition of clinical data and the evaluation of these. NJ participated in the conception and design, preparation of clinical grade TILs and carried out the ELIspot analyses. MD carried out some of the ELIspot analyses and all intracellular staining analyses and participated in analysis and interpretation of data. ÖM carried out the ELIspot analysis regarding mRNA transfected DCs and participated in analysis and interpretation of these data. RSA and SRH did the MHC multimer staining and participated in analysis and interpretation of these data. MHA and PtS participated in conception and design and interpretation of data. IMS participated in conception and design, analysis and interpretation of data and helped to draft the manuscript. All authors revised, read and approved the final manuscript.

## Supplementary Material

Additional file 1 Materials and methods on “Transfected dendritic cell based therapy for patients with breast cancer or malignant melanoma” (Engell-Noerregaard et al. trial ongoing, ClinicalTrials.gov ID: NCT00978913). Click here for file

Additional file 2 **Table S1.** Showing reactivity of the TIL infusion products. Click here for file

Additional file 3 **Figure S1.** Example of MHC multimer stainings. Click here for file
