# Supplementary material for: Adoptive cell therapy with autologous tumor infiltrating lymphocytes and low-dose Interleukin-2 in metastatic melanoma patients
Source: J Transl Med. 2012 Aug 21;10:169. doi: 10.1186/1479-5876-10-169 (PMC3514199; doi:10.1186/1479-5876-10-169)
Supplement: Additional file 1 — Materials and methods on “Transfected dendritic cell based therapy for patients with breast cancer or malignant melanoma” (Engell-Noerregaard et al. trial ongoing, ClinicalTrials.gov ID: NCT00978913). [file 1479-5876-10-169-S1.pdf]

Additional file 1:

Material and methods on "Transfected dendritic cell based therapy for patients with breast cancer or malignant melanoma" (Engell-Noerregaard et al, trial ongoing, www.clinicaltrials.gov ID: NCT00978913)

Plasmid constructs and in vitro transcription of mRNA

The plasmid pCI/hTERT/A102 (kindly provided by G Gaudernack, The Norwegian Radium Hospital, Oslo, Norway) has been described by Saebøe-Larssen et al[1]. The plasmid pSP73/p53/A64 was generated as described previously[2]. To generate pSP73/survivin/A64, we have subcloned the cDNA encoding survivin into pSP73/A64 (kindly provided by E Gilboa, University of Miami Miller School of Medicine, Miami FL, USA). The survivin cDNA fragment was excised from the pGEM-sig-survivin-DClamp plasmid (kindly provided by K Thielemans, Medical School of the Vrije Universiteit Brussel, Brussels, Belgium) using restriction site BamHI and inserted into pSP73/A64 that had been restricted with BamHI to generate pSP73/survivin/A64.

The generation of p53, survivin and hTERT mRNA was slightly modified compared to previously described[2]. In brief, prior to serving as DNA templates for in vitro transcription, the plasmids pSP73/p53/A64 and pSP73/survivin/A64 were linearized with SpeI, whereas the plasmid pCI/hTERT/A102 was linearized with MfeI, and all linearized plasmids were subsequently purified using Wizard DNA Clean-Up System (Promega, Madison WI, USA). The in vitro transcription was performed with mMESSAGE mMACHINE T7 Ultra kit (Ambion, Austin TX, USA) and mRNA was purified with MEGAclear kit (Ambion) according to the manufacturer's instructions. The mRNA length, concentration and purity were evaluated with the Agilent 2100 Bioanalyzer (Agilent Technologies, Palo Alto CA, USA), using RNA 6000 Nano LabChip Kit (Agilent Technologies) according to the manufacturer's instructions. Data analysis was performed with 2100 Bioanalyzer software (Agilent Technologies).

Vaccine preparation

The vaccine production was performed in a dedicated cell processing facility at Herlev Hospital using standardized, Danish Medicines Agency-approved protocols. For the generation of autologous monocyte-derived DCs, a concentrated leukocyte fraction was harvested by leukapheresis as described previously[3]. In brief, peripheral blood mononuclear cells (PBMC) from the leukapheresis product were incubated in a humidified incubator for 1 hour at 37°C to allow plastic adherence. The adherent cell fraction was used for DC culture by incubation for 5 days in X-VIVO 15 medium (Lonza, Basel, Switzerland) supplemented with 1% autologous heat-inactivated plasma, 1000 U/ml GM-CSF (Leukine) (Genzyme, Cambridge MA, USA) and 250 U/ml IL-4 (CellGenix, Freiburg, Germany). Maturation of DCs were performed on days 5–7 with TNF-α (1000 U/ml), IL-1β (1000 U/ml), IL-6 (1000 U/ml) (CellGenix) and 1 µg/ml PGE2 (ProstinE2, Pfizer Freiburg, Germany). Cells were harvested on day 7 and used for mRNA transfection. The transfection of mature DCs by electroporation was slightly modified compared to previously described[2]. Briefly, mature DCs[4] were washed twice, suspended in Opti-MEM medium (Invitrogen, Paisley, UK) and adjusted to a final cell density of 6.25×10^7 cells/ml. The cell suspension (800 µl) was preincubated in a 4-mm gap electroporation cuvette for five minutes on ice. Twenty µg of mRNA encoding p53, survivin or hTERT were transferred to the cuvette and DCs were pulsed using a BTX 830 square-wave electroporator (Harvard Apparatus, Holliston MA, USA). Electroporation settings were adjusted to a single pulse of 500 V and 2 ms. After electroporation, DCs were rested for 30 minutes in 37°C before frozen in aliquots of 1×10^7 DCs in 85% autologous serum, 10% DMSO (Wak-Chemie Medical GMBH, Steinbach, Germany) and 5% Glucosteril 40% (Fresenius, Albertslund, Denmark) using automated cryopreservation (Planer freezing unit; Planer, UK).

Microbiologic and endotoxin controls of DCs were performed on pre-freezing samples.

Reference List

1. Saeboe-Larssen, Fossberg, Gaudernack. mRNA-based electrotransfection of human dendritic cells and induction of cytotoxic T lymphocyte responses against the telomerase catalytic subunit (hTERT). J Immunol Methods 2002;259:191-203

2. Met, Balslev, Flyger, Svane. High immunogenic potential of p53 mRNA-transfected dendritic cells in patients with primary breast cancer. Breast Cancer Res Treat. 2011;125:395-406

3. Berntsen, Trepiakas, Wenandy, Geertsen, Thor, Andersen, et al. Therapeutic dendritic cell vaccination of patients with metastatic renal cell carcinoma: a clinical phase 1/2 trial. J Immunother 2008;31:771-80

4. Svane, Nikolajsen, Walter, Buus, Gad, Claesson, et al. Characterization of monocyte-derived dendritic cells maturated with IFN-alpha. Scand.J Immunol 2006;63:217-22
