# Supplementary material for: Adoptive cell therapy with autologous tumor infiltrating lymphocytes and low-dose Interleukin-2 in metastatic melanoma patients
Source: J Transl Med. 2012 Aug 21;10:169. doi: 10.1186/1479-5876-10-169 (PMC3514199; doi:10.1186/1479-5876-10-169)
Supplement: Additional file 2 — Table S1. Showing reactivity of the TIL infusion products. [file 1479-5876-10-169-S2.pdf]

Additional file 2:

Table showing reactivity of the TIL infusion products

**Patient number**	**Autologous tumor**	**Allogeneic HLA-A matched melanoma cell lines**								
		**909.04#**	**909.06#**	**131207#**	**FM55M1***	**FM6***	**FM3***	**909.11#**	**150710#**	**909.12#**
**1**	-	NR	0.11	0.42	0.41	NR	**28.82**	-	-	-
2	-	NR	NR	0.82	1.04	NR	0.89	NR	NR	-
3	-	-	-	NR	-	NR	-	NR	0.04	-
6	2.32	-	-	-	-	-	-	-	-	-
7	-	NR	NR	0.27	0.39	NR	0.19	-	-	0.1
**11**	**6.04**	-	-	-	-	-	-	-	-	-

All results are expressed as percentage of the double positive events (TNF-α/IFN-γ) in the $CD8^+$ gate

*long term cultured melanoma cell lines, # melanoma cell lines established from other patients with metastatic melanoma, these are short term cultured melanoma cell lines (<15-20 in vitro passages)

NR: no reactivity; -: not tested
