# Supplementary material for: Adoptive cell therapy with autologous tumor infiltrating lymphocytes and low-dose Interleukin-2 in metastatic melanoma patients
Source: J Transl Med. 2012 Aug 21;10:169. doi: 10.1186/1479-5876-10-169 (PMC3514199; doi:10.1186/1479-5876-10-169)
Supplement: Additional file 3 — Figure S1. Example of MHC multimer stainings. [file 1479-5876-10-169-S3.pdf]

Additional file 3

Examples of MHC multimer stainings

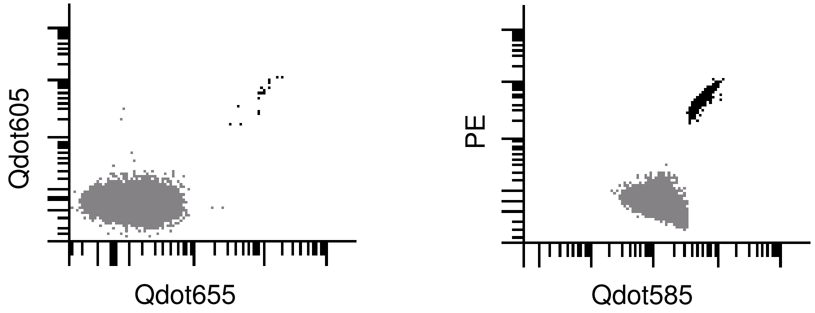

Patient 1: 0.01% gp100$_{KTW}$ Patient 6: 1.1% MART-1$_{ELA}$

Dot plots from 2 patients are shown as examples. MHC multimer positive populations representing antigen-specific T cells are shown in black and frequencies are given in percentage of CD8+ T-cells.

gp100$_{KTW}$ specific cells were stained with a combination of Qdot 655 and Qdot 605 labeled MHC multimers, and Mart-1$_{ELA}$ specific T cells were stained with a combination of Qdot585 and PE labeled MHC multimers.

Both X and Y-axes are biexponential display of the given fluorescence signal.
